# Hypoglycemia associated with fluoroquinolone: a pharmacovigilance analysis from 2014 to 2023 based on the FDA adverse event reporting system

**DOI:** 10.3389/fmed.2025.1583093

**Published:** 2025-05-02

**Authors:** Dan Li, Yuan Zhang, Yu Qi Wang, Lei Cheng, Xiao Dan Zhou, Jia Chen, Guo Wen Feng, Ya Ling Chen, Yun Chen, Zi Xue Xuan, Li Chen

**Affiliations:** ^1^Department of Pharmacy, Zhejiang Provincial People’ s Hospital Bijie Hospital, Bijie, China; ^2^Department of Pharmacy, University-Town Hospital of Chongqing Medical University, Chongqing, China; ^3^Department of Pharmacy, Chengdu Jinniu District People’s Hospital, Chengdu, China; ^4^Department of Pharmacy, Affiliated Hospital of Chengdu University, Chengdu, China; ^5^Department of Pharmacy, Center for Clinical Pharmacy, Cancer Center, Zhejiang Provincial People's Hospital (Affiliated People's Hospital), Hangzhou Medical College, Hangzhou, China; ^6^Department of Pharmacy, West China Second University Hospital, Sichuan University, Chengdu, China; ^7^Key Laboratory of Birth Defects and Related Diseases of Women and Children, Sichuan University, Ministry of Education, Chengdu, China

**Keywords:** FAERS, fluoroquinolones, disproportionality analysis, hypoglycemia, clinical characteristics

## Abstract

**Objective:**

With the increasing use of fluoroquinolones (FQs) for anti-infective therapy, the adverse events (AEs) caused by their collateral effects pose a key challenge in their clinical application. Hypoglycemia AEs are a type of AE linked to FQs and are commonly observed in real-world settings. Our objective was to provide a comprehensive analysis and summary of hypoglycemia AEs associated with FQs, specifically focusing on moxifloxacin (MOX), ciprofloxacin (CPR), and levofloxacin (LEV).

**Methods:**

Disproportionality analysis was used to assess the strength of the association between fluoroquinolone (FQ)-related hypoglycemia and potential safety signals, using data from the Food and Drug Administration (FDA) Adverse Event Reporting System (FAERS) covering quarters from 2014 Q1 to 2023 Q4. We utilized Standardized MedDRA Queries (SMQs) at the preferred term (PT) level to retrieve AE data from the FAERS reports. Following the removal of duplicate reports, a disproportionality analysis was conducted to identify potential safety signals associated with FQ-related hypoglycemia by calculating the reporting odds ratio (ROR). In addition, clinical characteristics, onset timing, oral and intravenous administrations, and serious outcomes related to FQ-associated hypoglycemia were further analyzed to provide a comprehensive understanding of the safety profile.

**Results:**

A total of 242,509 AE reports linked to FQs were detected, of which 16,306 indicated hypoglycemia signals. Among these cases, a higher percentage involved female patients than male patients (52.55% vs. 36.07%). The demographic analysis indicated that FQ-induced hypoglycemia was mainly concentrated in patients aged 18 ~ 64 years, with the mean age ranging from 51.14 to 57.39 years. Moreover, CPR had the most cases of hypoglycemia and related conditions (ROR 99.02, PRR 98.15, IC 35.66, EBGM 96.79), while LEV was relatively weakly associated with hypoglycemia (ROR 82.78, PRR 82.45, IC 27.89, EBGM 81.02). This study showed that all three FQs detected positive signals of hypoglycemia within 1 week. Oral fluoroquinolone-induced hypoglycemia had stronger signal strength than intravenous administration. Regarding the mortality and disability rates associated with hypoglycemia, MOX had the most cases of severe adverse drug events.

**Conclusion:**

FQ-induced hypoglycemia tends to occur early and can have serious consequences. Our preliminary findings provide a better understanding and emphasize the need for enhanced monitoring of potential hypoglycemia linked to FQ treatment.

## 1 Introduction

Fluoroquinolones (FQs) are commonly used antibacterial agents that are widely utilized across clinical departments due to their advantages of high oral bioavailability, excellent tissue penetration, and a long half-life (1, 2). FQs exert distinctive antibacterial effects by inhibiting DNA gyrase and topoisomerase IV, thereby directly inhibiting the synthesis of bacterial DNA (3). The most well-known and commonly used FQs are ciprofloxacin, norfloxacin, ofloxacin, levofloxacin, enrofloxacin, danofloxacin, and moxifloxacin, which have significantly enhanced antibacterial activity, especially against Gram-positive bacteria and anaerobes (4).

Although FQs are still considered to be mostly well-tolerated, there have been increasing concerns about their safety (5). Their most common adverse reactions (ADRs) are mild and reversible, such as diarrhea, nausea, or headaches, and lead to treatment discontinuation in less than 2% of patients (6). However, FQs are also associated with serious adverse events (AEs), such as hemolysis, renal failure, and hypoglycemia, which led to the withdrawal of temafloxacin (7). Trovafloxacin was removed from the market due to hepatotoxicity (8). FQ-induced anaphylactogenesis, tendonitis, and tendon rupture issues have been mostly reported in recent years (9, 10). In addition, post-marketing surveillance of adverse events (AEs) includes dysglycemia (hypoglycemia or hyperglycemia) (11). In 2018, the US Food and Drug Administration (FDA) issued a notice on its website, amending warnings and requesting that the drug inserts for systemic FQs be revised to strengthen the warning message that these drugs may cause significant reductions in blood glucose and psychiatric side effects (12).

In recent years, drug-associated glycemic abnormalities have been observed in patients during the clinical use of FQs, occurring in both diabetic and non-diabetic patients (13). The risk of possible glucose metabolism abnormalities induced by this class of drugs continues to attract clinical attention. Kennedy et al. (14) found that many patients on antibiotics, including FQs, are at risk of hypoglycemia when concurrently taking sulfonylureas or meglitinides. Patients on cefditoren, tigecycline, ertapenem, and clarithromycin may experience hypoglycemia even if they are not taking sulfonylureas or meglitinides. Currently, the exact mechanism by which antimicrobial drugs cause alterations in glucose metabolism is not fully understood (15). This effect may result from direct drug action on glucose metabolism or a combination of several factors.

Although systematic reviews and studies have examined fluoroquinolone-related hypoglycemia, the available data are outdated and limited in number (16). No existing literature has systematically compared FQs with the risk of hypoglycemia. This study aimed to assess the link between FQs and hypoglycemia and evaluate the safety of FQs for diabetic patients by examining reported dysglycemic side effects. To further explore this serious AE, we conducted a retrospective study utilizing large international pharmacovigilance databases to analyze AE reports associated with FQs (moxifloxacin MOX, ciprofloxacin CPR, and levofloxacin LEV), which are widely used in clinical practice, with a focus on hypoglycemia events reported through the FDA Adverse Event Reporting System (FAERS) database.

## 2 Methods

### 2.1 Study design and data sources

A retrospective disproportionality analysis reflecting the case/non-case study design was conducted to evaluate the potential association between FQs and a specific AE. The Food and Drug Administration Adverse Event Reporting System (FAERS) is a public database of AE reports submitted to the FDA, containing details on drugs such as name, active ingredient, administration route, role in the event, and reaction information (17). The FAERS data include drug details (name, active ingredient, administration route, and role in the event) and reaction information. Each report features a primary suspected drug along with associated adverse drug reactions. The database is updated quarterly, and users can freely download it in XML or ASCII format from the FDA website. All reports submitted to the FAERS from the first quarter (Q1) of 2014 to the fourth quarter (Q4) of 2023 were included in our study.

### 2.2 Data filtering

Each FQ antibiotic was identified in the FAERS by its generic and brand names listed in the Drugs@FDA Database (18). All reports with primary suspected drugs moxifloxacin, ciprofloxacin, and levofloxacin were filtered through fuzzy matching in the “drug name” field using MySQL to remove duplicate data. The AEs in REAC files are encoded by the preferred terms (PTs) in the Medical Dictionary for Regulatory Activities (MedDRA) version 27.1. To increase the accuracy of this study, data were cleaned by excluding duplicates and reports without age or sex information. Duplicate reports were defined as those having the same key information, such as age, sex, suspected drug, and adverse event reaction. We screened available Standardized MedDRA Queries (SMQs) using a “broad” version to investigate the association between FQs and hypoglycemia AEs. “Hypoglycaemic and neurogenic shock syndrome” and “Hypoglycaemic” were the two SMQs we selected to assess hypoglycemia. Furthermore, the time to onset of hypoglycemia and the proportion of serious outcomes caused by different FQs were calculated. The onset time was defined as the interval between EVENT_DT (date of AE occurrence) and START_DT (start date for FQ use). Reports with input errors (EVENT_DT earlier than START_DT), inaccurate date entries, and missing specific data were excluded (19).

### 2.3 Data extraction and descriptive analysis

The incidence of AEs cannot be calculated using the FAERS database, so the actual denominators are unknown (20). However, disproportionality analysis, an effective method in pharmacovigilance studies, was used to identify signals of disproportionate reporting for AEs related to FQ antibiotics in our study. Both Bayesian and frequentist methods were used to explore the association between FQ antibiotics and AEs, using the reporting odds ratio (ROR), the proportional reporting ratio (PRR), the information component (IC), and the empirical Bayes geometric mean (EBGM). Four algorithms were used. A positive signal of disproportionality was defined as a PRR of at least two, a chi-squared result of at least four, and an occurrence of three or more cases (17), with IC025>0 and EBGM>2.

**Figure 1:**
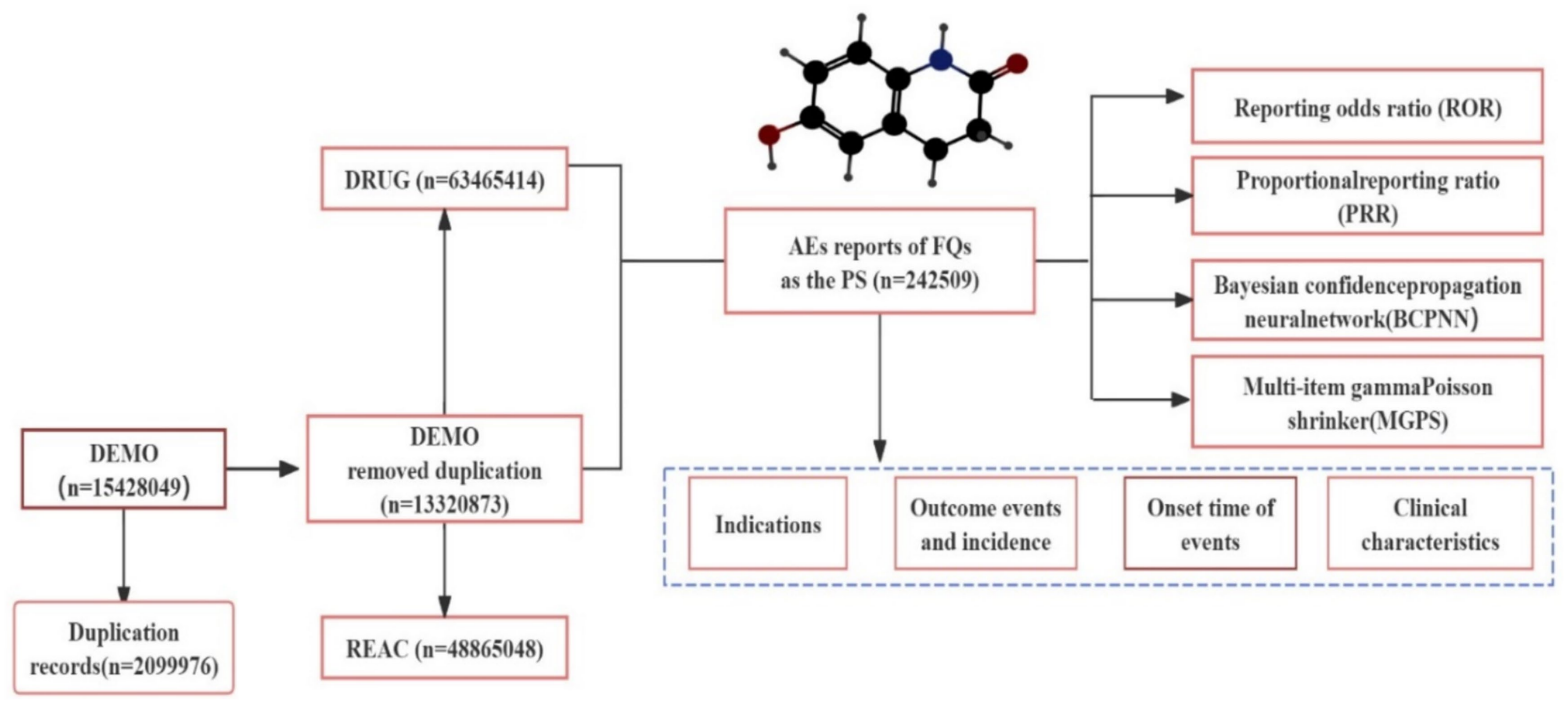
Process of selecting FQ-associated AEs from the FAERS database. FQs: fluoroquinolones, PS: primary suspected, AEs: adverse events.

Subsequently, we retrieved and described detailed information, including patient characteristics (sex, age), reporting area, indications, outcomes, and reporters. Notably, the total number of serious outcomes may exceed the total number of cases because some cases report more than one serious outcome. For example, a case may involve disability, hospitalization, and then death. The multistep process of data extraction, processing, and analysis is shown in Figure 1. All data processing and statistical analyses were performed using MYSQL 8.0, Navicat Premium 15, Microsoft EXCEL 2019, and GraphPad Prism 8 (GraphPad Software, CA, United States).

## 3 Results

### 3.1 Descriptive analysis

Our study analyzed 10 years of adverse drug event data from the FAERS, spanning from January 2014 to December 2023. We extracted a total of 13,320,873 reports from the FAERS database, with 242,509 identifying moxifloxacin (MOX), ciprofloxacin (CPR), and levofloxacin (LEV) as the “primary suspected” drugs. Among these, our analysis identified 16,306 cases related to hypoglycemia from the SMQs. Different FQs exhibited varying distributions of reported clinical characteristics, which are described in Table 1.

**Table 1:** Clinical characteristics of the reports with MOX-, CPR-, and LEV-associated hypoglycemia from the FAERS database (January 2014 to December 2023).

Characteristics	MOX (*N*, %)	CPR (*N*, %)	LEV (*N*, %)
Number of hypoglycemia events	1,530	9,056	5,720
Sex			
Female	800 (52.29)	4,871 (53.79)	2,898 (50.66)
Male	597 (39.02)	3,455 (38.15)	1,829 (31.98)
Unknown	133 (8.69)	730 (8.06)	993 (17.36)
Age (year)			
<18	16 (1.05)	111 (1.23)	101 (1.77)
18 ≤ and≤65	813 (53.14)	5,887 (65.01)	3,304 (57.76)
>65	466 (30.46)	1,979 (21.85)	1,945 (34.00)
Unknown	235 (15.36)	1,079 (11.91)	370 (6.47)
Mean age (year)	57.39	51.14	56.78
Indications			
Diabetes	1 (0.07)	11 (7.58)	4 (0.07)
Others	1,528 (99.87)	8,074 (89.16)	5,030 (87.94)
Product used for an unknown indication	1 (0.07)	971 (10.72)	686 (11.99)
Administration			
Oral	661 (43.20)	5,457 (60.26)	3,401 (59.46)
Intravenous	284 (18.56)	310 (3.42)	446 (7.80)
Serious Outcome			
Death (DE)	99 (6.47)	115 (1.27)	109 (1.91)
Life-threatening (LT)	137 (8.95)	291 (3.21)	107 (1.87)
Hospitalization—Initial or Prolonged (HO)	271 (17.71)	1,001 (11.05)	509 (8.90)
Disability (DS)	116 (7.58)	34 (0.38)	165 (2.88)
Congenital Anomaly (CA)	0 (0.00)	2 (0.02)	1 (0.02)
Required Intervention to Prevent Permanent Impairment/Damage (RI)	1 (0.07)	15 (0.17)	4 (0.07)
Other Serious Important Medical Events (OT)	672 (43.92)	1,263 (13.95)	339 (5.93)
Reporting region			
The United States	524 (34.25)	2,986 (32.97)	2,817 (49.25)
Germany	126 (8.24)	1,331 (14.70)	687 (12.01)
France	53 (3.46)	256 (2.83)	297 (5.19)
England	813 (53.14)	2,165 (23.91)	368 (6.43)
Other countries	0 (0.00)	2,318 (25.60)	1,551 (27.12)
Reported Person			
Health professional			
Physician (MD)	204 (13.33)	1,037 (11.45)	963 (16.84)
Pharmacist (PH)	83 (5.42)	455 (8.20)	363 (6.35)
Health Professional (HP)	291 (19.02)	958 (10.58)	397 (6.94)
Other health professional (OT)	317 (20.72)	1,259 (13.90)	749 (13.09)
Non-healthcare professional			
Consumer (CN)	530 (34.64)	4,545 (50.19)	2,473 (43.23)
Lawyer (LW)	30 (1.96)	59 (0.65)	0 (0.00)
Unknown	75 (4.90)	743 (8.20)	775 (13.55)

The most commonly reported hypoglycemia was associated with CPR (*n* = 9,056, 55.53%), followed by LEV (*n* = 5,720, 35.08%) and MOX (*n* = 1,530, 9.38%). Compared to CPR and LEV, MOX had a much lower frequency of hypoglycemia reports. Among these cases, a higher percentage involved female patients compared to male patients (52.55% vs. 36.07%). The demographic analysis revealed that the three FQs were mainly concentrated in patients aged 18 ~ 64 years, with the mean age of the patients ranging from 51.14 to 57.39 years. The most commonly reported therapeutic indication for all FQs was infections, while only a few diagnoses were related to diabetes. During the same study period, the hypoglycemia signal for MOX, CPR, and LEV was simultaneously detected and compared across different administration routes: oral and intravenous. It was observed that the hypoglycemia signals induced by the three FQs were more frequent and stronger when administered orally compared to intravenously.

Severe outcomes primarily consisted of death, life-threatening conditions, and prolonged hospitalization as a result of FQ-induced hypoglycemia. As for death, life-threatening condition, and prolonged hospitalization rates, MOX had the highest numbers (*n* = 99, 137, 271 6.47, 8.95, 17.71%), while CPR-induced hypoglycemia leading to death was the lowest (*n* = 115, 1.09%). LEV-induced hypoglycemia leading to life-threatening and prolonged hospitalization was the lowest. The vast majority of reports came from the United States and England.

### 3.2 Signal values associated with hypoglycemia

**Figure 2:**
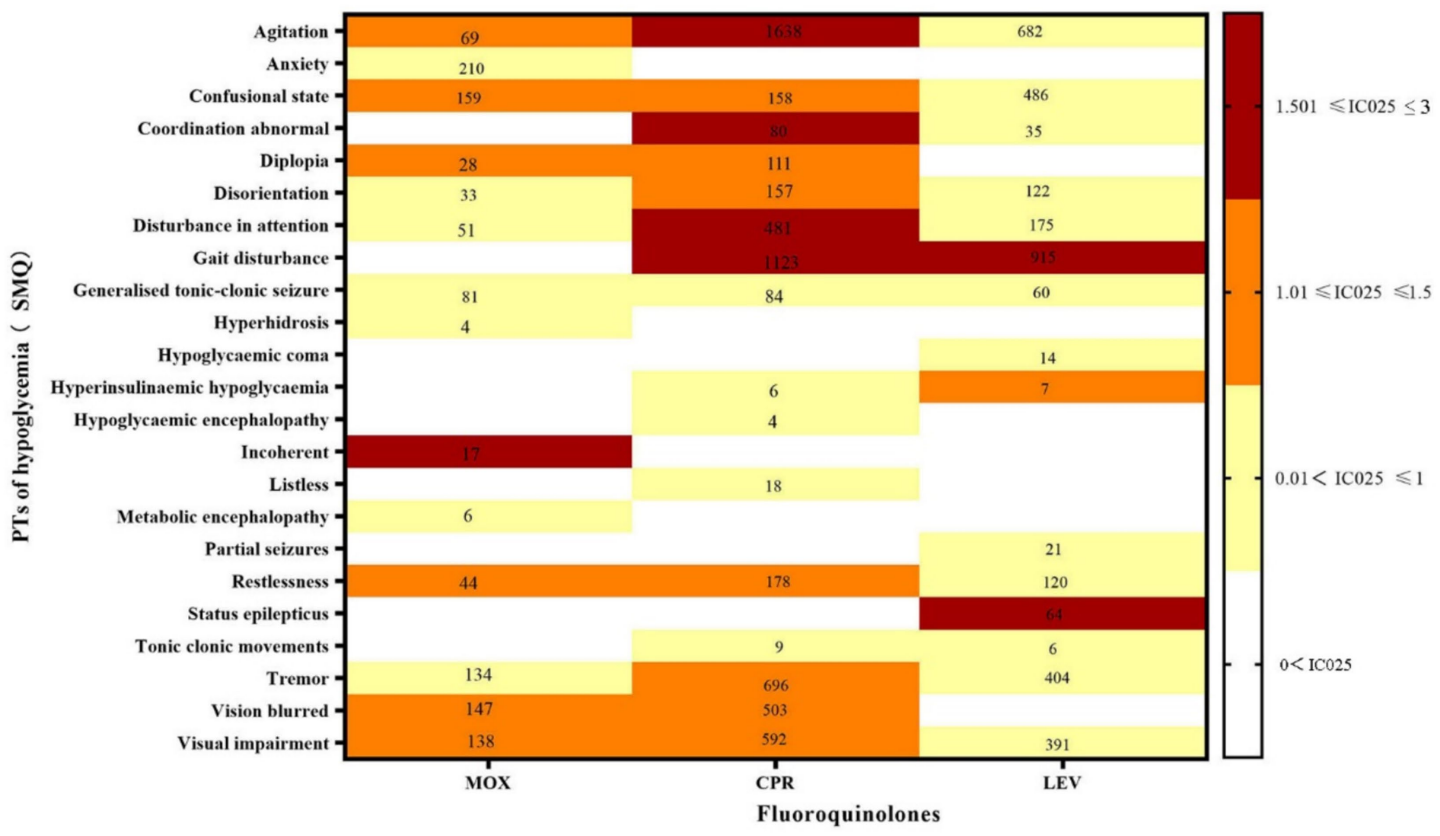
Hypoglycemia signal profiles of different FQ strategies. PTs: preferred terms; IC025: the lower end of the 95% confidence interval in the information component. The numbers in the blocks represent the reported values for each combination of target FQs and PTs. The color of the blocks represents relevance.

**Figure 3:**
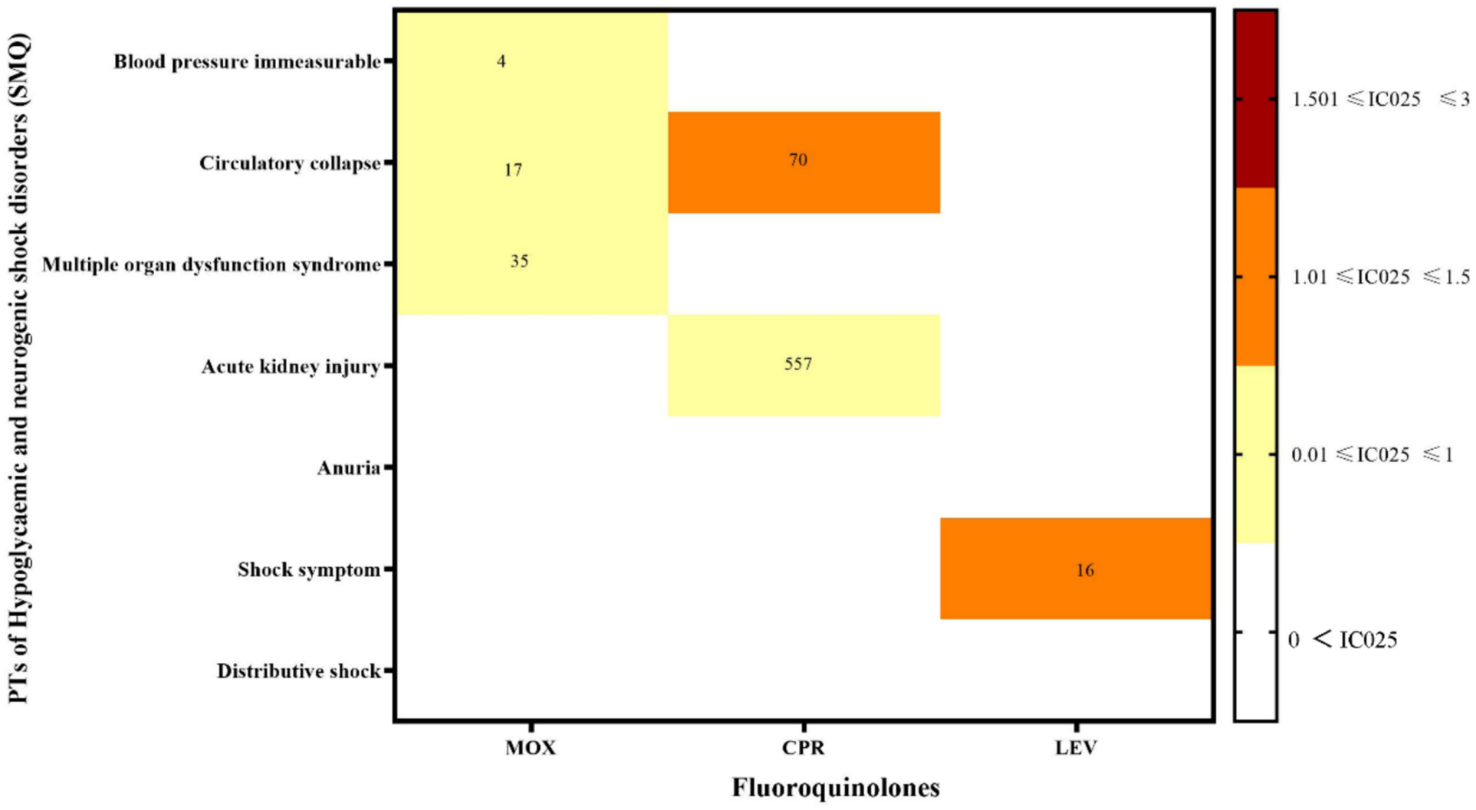
Hypoglycemic and neurogenic shock disorder signal profiles of the different FQ strategies. PTs: preferred terms; IC025: the lower end of the 95% confidence interval of the information component. The numbers in the blocks represent the reported values for each combination of target FQs and PTs. The color of the blocks represents relevance.

The hypoglycemia signals for the three FQs based on the criteria of the four algorithms are summarized in Table 2. CPR had the strongest statistical association with hypoglycemia, with the highest positive signal values (ROR 99.02, PRR 98.15, IC 35.66, EBGM 96.79), while LEV was relatively weakly associated with hypoglycemia (ROR 82.78, PRR 82.45, IC 27.89, EBGM 81.02), exhibiting the lowest signal strength. Statistically, hypoglycemia AEs were notable, defined by 30 PTs that could be classified into two categories by the SMQs: “hypoglycaemic” and “hypoglycemic and neurogenic shock.” To investigate the association between the various FQs and specific hypoglycemia AEs, we explored the spectrum of hypoglycemia for each; details are shown in Figures 2, 3. CPR showed the widest spectrum of hypoglycemia AEs, with 18 PTs detected as signals, ranging from IC025 = 0.19 (hypoglycemic encephalopathy) to IC025 = 1.85 (agitation). For LEV, a total of 15 PTs were observed as signals, with signal values ranging from IC025 = 0.13 (tonic–clonic movements) to IC025 = 1.59 (gait disturbance). However, the drug with the fewest PTs was MOX, with signal values ranging from IC025 = 0.01 (hyperhidrosis) to IC025 = 1.77 (incoherent). In particular, agitation, confusional state, disorientation, generalized tonic–clonic seizure, restlessness, tremor, and visual impairment showed significant associations with all three FQs. Focusing on hypoglycemic and neurogenic shock syndrome, CPR had the highest number of reported cases (*N* = 627), and the main manifestations were acute kidney injury and circulatory collapse (as shown in Figure 3).

**Table 2:** Signal detection for MOX-, CPR-, and LEV-associated hypoglycemia.

	*N*	ROR (95% CI)	PRR (χ^2^)	IC (IC025)	EBGM (EBGM05)
Hypoglycemia					
MOX	1,177	97.74 (60.72–173.97)	97.11 (2595.48)	32.81 (15.89)	96.81 (60.07)
CPR	6,890	99.02 (70.40–156.42)	98.15 (16991.93)	35.66 (21.80)	96.79 (68.83)
LEV	3,518	82.78 (54.55–135.14)	82.45 (5232.3)	27.89 (14.17)	81.02 (53.51)

*N, number of adverse event reports; ROR, the reporting odds ratio; PRR, the proportional reporting ratio; IC, the information component; EBGM, the empirical Bayes geometric mean; CI, confidence interval; 95% CI, two-sided for ROR, χ2, chi-squared; IC025 and EBGM05 are lower one-sided for IC and EBGM.*

### 3.3 Time to onset of FQ-associated hypoglycemia

**Figure 4:**
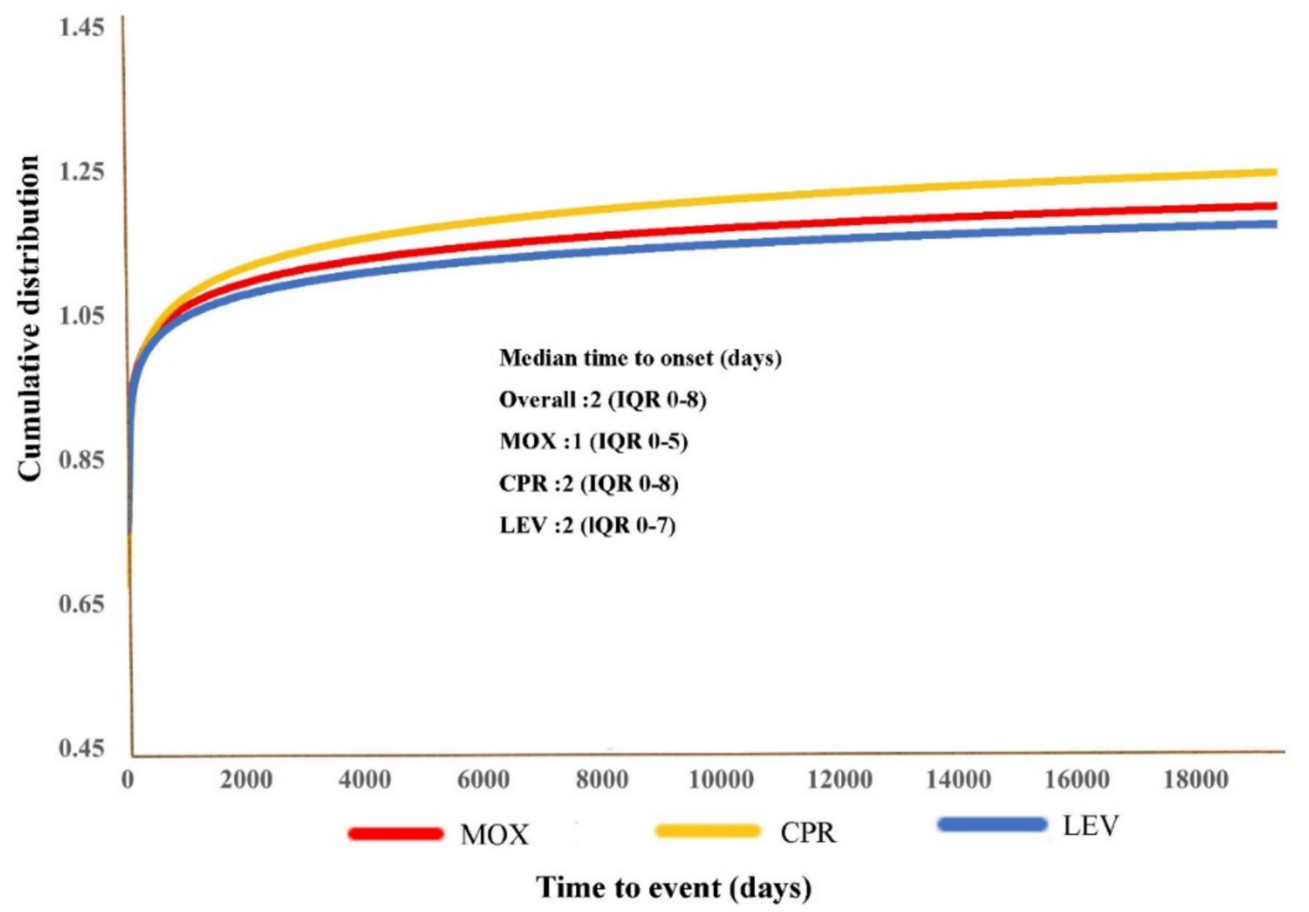
Cumulative distribution curves demonstrating the onset time of FQ-related hypoglycemia adverse events (AEs) after treatment with the different FQs.

A total of 10,888 MOX-, CPR-, and LEV-associated hypoglycemia reports with onset times were analyzed. The time to onset of hypoglycemia for each FQ is shown in Figure 4. Of all the validated reports, the median time to onset of hypoglycemia AEs for the overall FQs was 2 days (IQR = 0–8 days), with 74.53% of the AEs occurring within the first 7 days. MOX-related hypoglycemia had the shortest median onset time of 1 day (interquartile range [IQR] 0–5).

## 4 Discussion

As described in the Introduction section, previous studies on FQ-related adverse events (AEs) have primarily focused on anaphylactogenesis, tendonitis, and tendon rupture. On 10 July 2018, the FDA issued a request to update the drug insert for systemic fluoroquinolone antibiotics to emphasize the risks of significant reductions in blood glucose levels and psychiatric side effects. There have been few studies on the mechanism of FQ-induced hypoglycemia AEs. Some scholars have found (21) that FQs can induce glycemic aberrations by enhancing pancreatic cells’ insulin secretion and by interacting with antidiabetic agents via the inhibition of cytochrome P450 enzymes. Atsunori et al. (22) found that the stimulation of insulin secretion through the inhibition of pancreatic beta-cell K (ATP) channels underlies the hypoglycemia caused by certain FQs.

Therefore, we examined reports of FQ-induced hypoglycemia AEs using the entire FAERS database as a comparator, detailing the real-world reporting of such AEs and the corresponding clinical characteristics. Using disproportionate analysis methods, we identified two hypoglycemia AEs highly associated with FQ treatment and analyzed the data on their variability.

Using extensive real-world FAERS data, we found that CPR was linked to the highest risk of hypoglycemia. Although MOX was reported less frequently, a significant adverse drug event signal for hypoglycemia was still observed. This was because the FAERS database included at least 1,701 MOX AE reports, compared to 5,720 for LEV and 9,506 for CPR. This discrepancy could be due to CPR and LEV being the most frequently used FQs in clinical settings (23). In addition, we found that infections were the primary therapeutic indication for all FQs, with few diagnoses related to diabetes. This aligns with previous studies (24, 25). A randomized, double-blind, crossover study (26) demonstrated that hypoglycemia was reported in 2,179 patients who received fluoroquinolones across 11 studies with a total of 6,208 patients. Dysglycemia was not generally associated with diabetes mellitus per se.

In this study, female patients had a slightly higher incidence of FQ-related hypoglycemia compared to male patients. The finding is consistent with that of a previous study that assessed the risk of FQ-associated hypoglycemia (27). Moreover, the average age of patients with hypoglycemia ranged from 51.14 to 57.39 years. Severe outcomes included death, life-threatening conditions, and prolonged hospitalization as a result of FQ-induced hypoglycemia. MOX had the highest number of severe AE cases. This finding aligns with earlier reports on risk factors for FQ-induced hypoglycemia (28). A cohort study (29) demonstrated that patient factors associated with FQ-related hypoglycemia included older age, female sex, Black or Hispanic ethnicity, higher comorbidity, and a history of prior hypoglycemic episodes. In addition, 28.3% of patients prescribed a sulfonylurea filled a prescription for LEV or CPR antimicrobials, which were associated with 13.2% of all hypoglycemia events in patients taking sulfonylureas, occurring within 14 days of antimicrobial exposure and leading to hospitalization or emergency department visit. Based on the results from our FAERS data and the literature, clinicians should be particularly cautious when prescribing fluoroquinolones to the elderly, especially those with comorbidities or those taking sulfonylureas.

According to our study, an association was observed between hypoglycemia and FQs, although it was only supported by case reports (30). The consequences of hypoglycemia include acute and long-term cognitive changes, cardiac arrhythmias, myocardial infarction, serious falls, frailty, and death, often resulting in hospitalization (31). This is why the signals “vision blurred,” “gait disturbance,” “agitation,” “disorientation,” and “disturbance in attention” were detected through further data mining. It is worth noting that CPR was the only drug linked to hypoglycemic encephalopathy in the hypoglycemia SMQ, with all reported cases involving male patients over 70 years old. Hypoglycemia can cause nerve cell damage in the cerebral cortex and harm intracranial smooth muscle and vascular endothelial cells, leading to blood vessel wall swelling and issues related to cerebral circulation (32). Clinically, symptoms include central or focal neurological signs such as headache, dizziness, coma, convulsions, hemiplegia, urinary incontinence, brainstem signs, tetraplegia, paraplegia, and paroxysmal choreoathetosis (33). However, MOX-induced hypoglycemia AEs were the least reported but still showed signals of metabolic encephalopathy, anxiety, and other symptoms, which were not detected with the other two FQs and were not included in the specification for moxifloxacin. A meta-report (34) revealed that metronidazole and FQs were the most commonly reported antibiotics linked to brain lesions. Therefore, blood glucose levels should be regularly monitored in non-diabetic elderly individuals taking CPR and MOX. For elderly diabetic patients, close monitoring is essential, and insulin or oral hypoglycemic doses should be adjusted promptly if they have infections or reduced food intake to prevent metabolic/hypoglycemic encephalopathy.

In addition, there have been few reports of hypoglycemic and neurogenic shock induced by FQs, mainly manifesting as immeasurable blood pressure, circulatory collapse, acute renal failure, multiple organ dysfunction syndrome, and other complications. Among these, MOX had the most PTs, while CPR had the highest number of cases. The most frequently reported ADRs of MOX was multiple organ dysfunction syndrome. As described in a case report (35), a 76-year-old woman with chronic obstructive pulmonary disease presented with dyspnea, fever, and productive cough. Intravenous antibiotic therapy with MOX was initiated, but soon thereafter, the patient developed septic shock with hypotension, acute respiratory failure, and renal failure. Xiao et al. (36) found that MOX-induced multiple organ dysfunction may be related to mutations in several genes involved in drug metabolism pathways. Clinicians should closely monitor blood glucose when using MOX. If recurrent hypoglycemia occurs, organ function should be checked to prevent severe hypoglycemia and neurogenic shock.

A cohort study (37) on diabetes and the risk of hypoglycemia after hospital discharge in patients with acute kidney injury (AKI) found that among 295,279 eligible patients with diabetes who had an index hospitalization during the study period, 73,761 (25%) experienced hospital-acquired AKI. This may explain why the “acute kidney injury” signal was detected during CPR data mining. More concerning is that patients with AKI may experience blood glucose metabolism disorders or hypoglycemia due to slow drug metabolism when using glucose-lowering drugs (38). Therefore, individuals with renal diseases or diabetes should closely monitor their blood glucose levels when taking FQs or consider avoiding MOX.

Hypoglycemia does not necessarily occur during or immediately after medication. Indeed, a significant number of AEs occurred within a few days or a week of FQ initiation, and some even emerged after several months, based on our FAERS results. Reports (39, 40) have indicated that FQ-induced hypoglycemia typically occurs within a few days of starting treatment. Therefore, it is suggested that clinical medical staff should focus on the occurrence of hypoglycemia AEs within the first week after medication during the follow-up period and take appropriate treatment measures when necessary. In addition, in our analysis of the routes of administration for the three FQs, we identified a stronger signal for adverse hypoglycemic events associated with oral FQs, indicating a higher risk of these events. To date, few studies have demonstrated the relationship between the route of administration of FQs and the occurrence of hypoglycemia AEs, which may be caused by the accumulation of drugs in patients after intravenous infusion and continuous oral sequential therapy.

### 4.1 Strengths and limitations of the study

The FAERS is a public, accessible, and free database in the United States that contains tens of millions of AE reports voluntarily submitted by healthcare professionals, patients, manufacturers, and other stakeholders. It is designed to facilitate the FDA’s surveillance of safety issues related to post-market drugs and biologicals. We used four distinct analytical approaches to investigate FQ-induced hypoglycemia AEs, striving to minimize the occurrence of spurious associations. Moreover, when analyzing the timing of hypoglycemia AEs and serious clinical outcomes, we selected the data used in the analyses to ensure maximum consistency and accuracy, thereby providing a reliable guide for the clinical use of FQs in monitoring hypoglycemia AEs and informing subsequent mechanistic studies. Despite our efforts to enhance data reliability, the findings remain inevitably biased due to the inherent autonomy in the data reporting process. Adverse drug event signal detection addresses post-marketing specification issues, such as lag, uncertainty, and incompleteness, and enhances drug safety assessment by analyzing extensive AE spontaneous report databases (41). Our study has some notable limitations. First, the FAERS data rely on the reporter’s skills, which can result in biased reports due to missing, inadequate, or selective information. Second, controlling for confounding factors such as age, dose, comorbidities, and drug combinations that may influence adverse events is challenging. Finally, the FAERS lacks morbidity data due to insufficient information on all fluoroquinolone users, making it impossible to compare hypoglycemia incidence among different FQs.

## 5 Conclusion

Through a pharmacovigilance study, we conducted an exhaustive and systematic extraction and analysis of reports detailing hypoglycemia AEs linked to the administration of FQs. Among these FQs, ciprofloxacin (CPR) posed the greatest risk for hypoglycemia events, while MOX exhibited the fewest reported cases. Furthermore, we explored the onset time and serious outcomes associated with FQ-induced hypoglycemia, providing valuable insights for clinical practice and also for drug monitoring efforts to some extent.

Despite several limitations in the analysis of the FAERS database, the preliminary findings from this study provide a solid foundation for understanding potential hypoglycemia AEs that may occur with FQ treatment. These insights can assist clinicians by directing their attention to these rare yet significant AEs, enabling them to implement targeted interventions to optimize patient risk management.

## Data Availability

The datasets presented in this study can be found in online repositories. The names of the repository/repositories and accession number(s) can be found in the article/Supplementary material.
